# Characterization of monoclonal antibodies against Muscovy duck reovirus σB protein

**DOI:** 10.1186/1743-422X-7-133

**Published:** 2010-06-23

**Authors:** Ming Liu, Xiaodan Chen, Yue Wang, Yun Zhang, Yongfeng Li, Yunfeng Wang, Nan Shen, Hualan Chen

**Affiliations:** 1 National Avian Influenza Reference Laboratory, Animal Influenza Laboratory of the Ministry of Agriculture, Harbin Veterinary Research Institute, CAAS, Harbin 150001, China; 2 National Key Laboratory of Veterinary Biotechnology, Harbin Veterinary Research Institute, CAAS, Harbin 150001, China

## Abstract

**Background:**

The σB protein of Muscovy duck reovirus (DRV), one of the major structural proteins, is able to induce neutralizing antibody in ducks, but the monoclonal antibody (MAb) against σB protein has never been characterized.

**Results:**

Four hybridoma cell lines secreting anti-DRV σB MAbs were obtained, designated 1E5, 2F7, 4E3 and 5D8. Immunoglobulin subclass tests differentiated them as IgG2b (1E5 and 4E3) and IgM (2F7 and 5D8). Dot blot and western blotting assays showed that MAbs reacted with His-σB protein in a conformation-independent manner. Competitive binding assay indicated that the MAbs delineated two epitopes, A and B of σB. Immunofluorescence assay indicated that the four MAbs could specifically bind to Vero cells infected with DRV and σB was distributed diffusely in the cytoplasma of infected cells. MAbs had universal reactivity to all DRVs tested in an antigen-capture enzyme-linked immunosorbent assay.

**Conclusion:**

Results of this research provide important information about the four monoclonal antibodies and therefore the MAbs may be useful candidate for the development of a MAb capture ELISA for rapid detection of DRVs. In addition, it showed that the σB protein was located in the cytoplasma of infected cells by immunofluorescence assay with MAbs. Virus isolation and RT-PCR are reliable way for detection of DRV infection, but these procedures are laborious, time consuming, and requiring instruments. These obvious diagnosis problems highlight the ongoing demand of rapid, reproducible, and automatic methods for the sensitive detection of DRV.

## Background

The Muscovy duck reovirus (DRV) consists 10 segments of double-stranded RNA (dsRNA) packaged into a non-enveloped icosahedral double-capsid shell [1,2]. The genomic segments can be separated into three size classes: large (segments L1-L3), medium (segments M1-M3), and small (segments S1-S4) [1,3,4]. DRV is an important poultry pathogen associated with a variety of clinical syndromes in ducks [5-7]. DRV could cause high morbidity and up to 50% mortality in ducklings [3,8] and recovered ducks are markedly stunted in growth.

All avian reovirus (ARV) encoded proteins including at least 10 structural proteins (λA, λB, λC, μA, μB, μBC, μ1C σC, σA, and σB) and 4 nonstructural proteins (μNS, P10, P17, and σNS). The σB protein of DRV encoded by S3 gene segment is structurally related to the σ3 protein of mammalian or σB of ARV [9-12] and may be functional related. The σB protein is a major constituent of the outer capsid and, like σC, is exposed to the surface of the virion [2]. σB protein induce group-specific neutralizing antibody, while protein σC induces type-specific neutralizing antibodies [4].

Many methods have been developed for the diagnosis of DRV or ARV infections. Agar gel immuno-diffusion test (AGID)[13,14], Serum neutralization test (SN) [3,15], and enzyme-linked immunosorbent assay (σB-σC-ELISA) [12,16] are designed to detect antibodies to DRV or ARV. Immunofluorescent staining [6] offers the direct detection of viral antigens in tendon tissues. Recently, the one step RT-PCR method for the detection of ARV, DRV and goose reovirus (GRV) RNA from the cell culture and specimens [17] has been developed, providing a sensitive tool for diagnosis of different bird species reovirus infections. However, these methods possess some general problems, as they are time-consuming and labor-intensive, require sophisticated instruments.

In this study, four monoclonal antibodies (MAbs) directly against bacterially expressed σB protein of DRV were produced and characterized. Due to its universal reactivity to DRVs, it is an ideal candidate for use in an antigen-capture enzyme-linked immunosorbent assay (ELISA) for clinical diagnosis.

## Methods

### Cell and virus

The DRV S12 and several field isolates (S14, 044, F, and C4 strains) were used in this study [17]. All the DRV isolates were propagated in duck embryo fibroblasts (DEF) or Vero cells. The supernatant obtained by centrifugation of these lysates was treated with 1% Triton X-100 and used as a crude antigen for the antigen-capture ELISA.

### Antigen preparation

σB protein used for the production and characterization of MAbs were synthesized in *Escherichia coli *BL21 (DE3) as described before [12]. The expressed His-σB and 6.7 His proteins were purified by using Ni-NTA kit (Qiagen, Valencia, CA). This 6.7 kDa protein was used as a negative control during screening specific antibodies to σB in an ELISA.

### Monoclonal antibodies production

BALB/C mice were immunized intraperitoneally with 30 μg of antigens containing σB fusion protein in complete Freund's adjuvant and boosted twice with the same amount of antigens in incomplete Freund's adjuvant at 2 weeks intervals. Six weeks after the initial immunization and 4 days before the mice were sacrificed for the preparation of hybridoma, final boost was carried out in the same route with 30 μg of the same antigens. MAbs were produced using techniques similar to that described previously [18]. Briefly, spleens were removed from mice immunized with antigens containing σB as described above. Splenocytes were fused with NS1 myeloma cells. Hybridoma cell lines secreting antibodies against σB were screened and subcloned at least three times by a limiting dilution method and ascitic fluids were prepared with the cloned hybridoma in BALB/C mice.

### Serological screening

Hybridoma culture supernatants or mouse ascetic fluids were screened for antibodies in an indirect ELISA as described for antibody binding assay. Antibodies that bound to σB protein but failed to bind 6.7 kDa protein were considered to be positive to σB.

### Isotyping

Isotypes of the produced MAbs were determined by using Mouse Immunoglobulin isotyping kit (Zymed Laboratories, Inc.) according to the manufacture's instruction.

### Western blot assay and Immuno-dot binding assay

To examine whether S12 σB MAbs recognize the linear epitope of S12 σB protein, Western blotting was used to examine the binding ability of MAbs to denatured His-σB proteins. Purified His-σB protein was subjected to 10% SDS-PAGE and transferred to nitrocellulose membranes. The membranes were probed with different MAbs followed by a secondary HRP-conjugated goat anti-mouse antibody (KPL, MD, USA). His-σB and His proteins (as negative control) were used for dot blotting assays. Approximately 1 μg antigen was diluted with TNE buffer and spotted onto nitrocellulose membrane. The membranes were probed with the same MAbs as for western blot.

### Detection of native σB protein by immunofluorescence assay

Vero cells were infected with DRV S12 strain (10 M.O.I.) and incubated at 37°C for 24 h. The cells were fixed with cold methanol for 10 min and then probed with different anit-σB MAbs and negative normal mouse serum for 1 h at 37°C. Bound antibodies were visualized using fluorescent conjugated antibodies against mouse IgG (1:500 dilutions) under a fluorescence microscope.

### Coupling of horseradish peroxidase to monoclonal antibodies

Immunoglobulin fractions were isolated from ascetic fluids by precipitation at 4°C with an equal volume of saturated ammonium sulfate (pH 7.0), and then purified using an affinity column of protein G-agarose (Boehringer Mannheim). Antibodies were coupled to HRP by the periodate method [19] and stored at -20°C.

### Determination of MAbs titers

The titres of MAbs were determined using an ELISA. Expressed His-σB protein was coated into each well of plates with 0.1 μg at 37°C for 2 h. The plates were washed three times with washing buffer (0.01 M phosphate-buffered saline, pH 7.2, 0.05% Tween 20) and blocked with 100 μl TNE buffer containing 2.5% bovine serum albumin. After washing, two-fold serial dilutions of 1 μg/ml uncoupled or HRP-coupled MAbs were added and incubated for 1 h. For uncoupled MAbs, an additional 50 μl HRP-coupled goat anti-mouse antibodies were added. The absorbance value was read at 405 nm with a Microplate Reader (BIO-RAD). The level of binding for the relative activity was measured from the resulting dose-response curve.

### Antibody binding assay

To carry out the competitive binding assay, the amount of binding in the ELISA was determined for all MAbs uncoupled with HRP or coupled [20]. Briefly, for HRP-unconjugated MAb determination, ELISA plates were coated with 0.1 μg purified σB per well at 37°C for 2 h. After washing, 100 μl of TNE buffer containing 2.5% bovine serum albumin was added to each well to saturate all unbound sites. After washing, 100 μl of purified MAb serially diluted with TNE buffer containing 1% bovine serum albumin was added and incubated for 2 h at 30°C. After washing, 50 μl of a 1:500 dilution of HRP-conjugated goat anti-mouse IgG serum was added and incubated for another 1 h. The enzymatic activity was determined after 20 min of incubation by the addition of 30 ml of 1% sodium azide. The absorbance was measured at 405 nm. For HRP-conjugated MAb determination, the same procedures were carried out except that HRP conjugated MAbs were directly added to the antigen coated plates without using the HRP-conjugated goat anti-mouse antiserum. The level of maximum binding for the relative activity measurement and the MAb concentration at which 50% binding occurred were obtained from the resulting dose-response curve.

### Competitive binding assay

Competitive binding assay were similarly to the procedures described above, except for a mixture of the HRP-conjugated MAbs at twice the concentration, giving half maximal binding. Unconjugated, competing antibodies at different concentrations were also added simultaneously. The competition between two MAbs against the same site was related to their relative avidities and concentrations. A spectrum of dose-related interference was tested. Non-specific binding without antigens was used to represent the background. The degree of competitive binding was measured from the absorbance at 405 nm in the presence or absence of unconjugated competing antibodies. Competition was rated as strong (++) if it was more than 60%, significant (+) if it was more than 30%, and negative (--) if it was less than 30%.

### Cross-reactivity of the MAbs to heterologous DRV strains

To study the monoclonal antibodies for their cross-reactivity with various DRV strains in an antigen-captured enzyme-linked immunosorbent assay (ELISA), four DRV field isolates were tested. MAbs (1E5 and 2F7) were used to prepare an antigen-capture ELISA and compared with the polyclonal antibody against DRV S12. Briefly, 100 μl mouse anti-σB polyclonal antibodies (1:200) were coated onto ELISA plates. After washing and blocking, 100 μl of cell extracts of DEF infected with each DRV isolates or from mock-infected cells were added and incubated for 1 h at 37°C. For the reaction of MAbs, 50 μl of HRP-conjugated MAbs (1:1000) were added as the primary antibody. To determine if σB present in each cell extracts from DEF/Vero infected with each DRV isolates was captured by anti-σB antiserum, duck antiserum against DRV S12 and HRP-coupled goat anti-duck antiserum were used as a primary and secondary antibodies, respectively. Absorbance was measured at 405 nm. Binding to the heterologous virus is expressed as percentage by taking the absorbance obtained with DRV S12 in the reaction as 100. Binding was rated as strong if it was more than 50%, significant if it was 25-50%, and negative if it was less than 25%.

## Results

### Production and general characterization of MAbs

At 3 weeks after cell fusion, the hybridoma cell lines secreting anti-σB antibody were screened by ELISA. Four MAbs directed against σB were selected for subcloning at least three times using the limiting dilution method. Hybridomas were selected to produce MAbs in mice and the ascitic fluids were used for further characterization. The isotypes of MAbs were IgG2b (1E5 and 4E3) and IgM (2F7 and 5D8), respectively. Concentrations of immunoglobulin ranged from 0.35 to 15.76 μg/ml.

### Effect of denaturation of σB on MAb recognition

**Figure 1:**
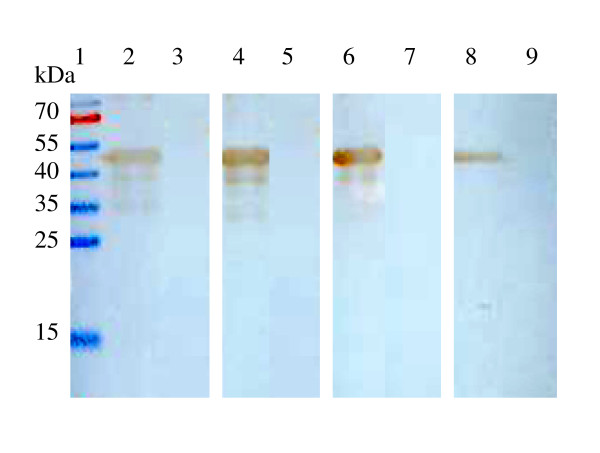
**Reactivity of S12 σB MAbs to *E coli *expressed pET30-σB and pET30a vector**. Lane 2, 4, 6, and 8 *E. coli *expressed pET30-σB; lane 3, 5, 7, and 9 *E.coli *expressed pET30a vector; lane 2 and 3 MAb 1E5; lane 4 and 5 MAb 4E3; lane 6 and 7 MAb 2F7; lane 8 and 9 MAb 5D8, respectively.

**Figure 2:**
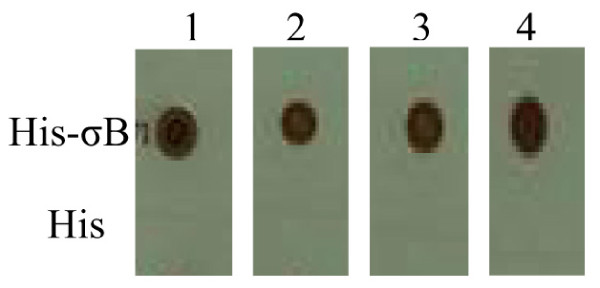
**Dot blotting assays of MAbs to His-σB and His proteins**. Lane 1, MAb 1E5; lane 2, MAb 4E3; lane 3, MAb 2F7; lane 4, MAb 5D8.

The expressed His-σB proteins were denatured by boiling in SDS and 2-mercaptoethanol, and subjected to western blotting; four MAbs still recognized them (Fig. 1). To determine if a native structure of σB is required for antibody binding, the native antigens containing σB was examined by using immuno-dot binding assay. All MAbs recognized the native structure of σB in TNE buffer (Fig. 2), but did not react with His proteins.

### Detection of native σB protein by immunofluorescence assay

**Figure 3:**
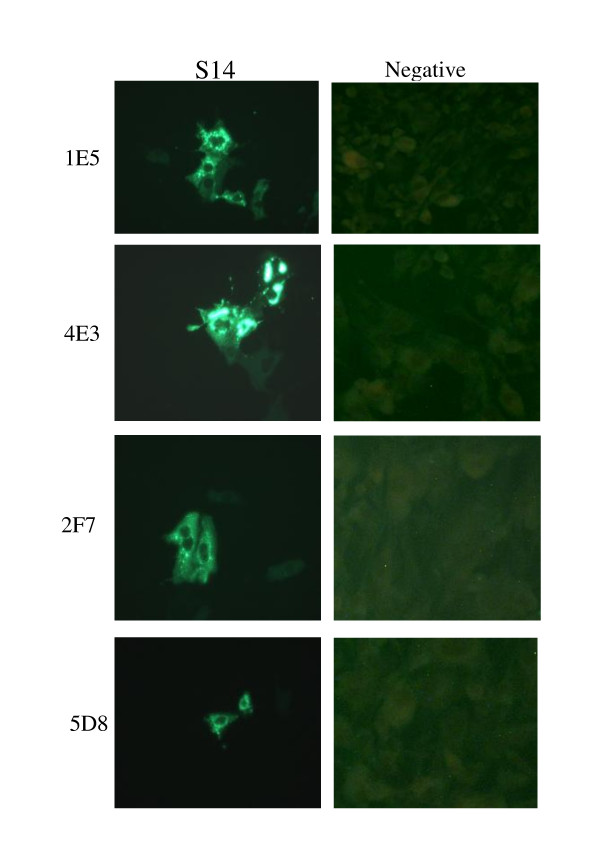
**Detection of S12 σB protein by indirect immunofluorescence assay on Vero cells infected with S12**. No special fluorescence was found on normal cell (400 ×).

Immunofluorescence assay was performed on S12 infected Vero cells to assess whether the S12 σB MAbs recognize the native-form of σB protein. Four σB MAbs strongly reacted with S12 infected cells, whereas uninfected cells showed no reaction (Fig. 3). The fluorescence signals of the MAbs were predominantly visualized in the cytoplasm of S12 infected cells. This indicated that all MAbs were able to detect native-form σB protein in S12 infected cells.

### Avidity of MAbs to σB

The amount of MAbs bound to the σB proteins can be quantified within the linear range of absorbance. This offers an estimation of the relative avidity of MAbs for their binding proteins. Binding degrees of MAbs to His-σB using ELISA titration indicated that all MAbs saturated at dilutions from 10^-1 ^and 10^-1.8^. Four MAbs retained their binding capacity after coupling to HRP, and the dilution range of saturation was 10^1 ^to 10^2^. No apparent saturation appeared in the remaining HRP-MAbs (data not shown).

### Mapping of the epitopes

The proper concentrations for the competitive binding assay were determined using dose-response curves plotted for unconjugated and HRP-conjugated MAbs (data not shown). Each of the four MAbs was used both as a competitor and as HRP-conjugated probe. The percentage of competition was normally 100% in the presence of a saturating unlabeled homologous antibody. Two distinct epitopes on σB were found and designated A and B (Table 1). 1E5, 4E3, and 5D8 all belong to epitope A, while 2F7 belong to epitope B.

**Table 1:** Results of competitive binding assay between MAbs against σB protein

Competitor	HRP-labeled MAbs			
	
	1E5	4E3	58	2F7
Epitope A				
1F5	++	++	++	--
4F3	++	++	++	--
5D8	++	++	++	--
Epitope B				

2F7	--	--	--	++

### Detection of DRV σB antigens

MAbs 1E5 and 2F7, representing MAbs recognizing epitopes A and B, respectively, were selected for testing their cross-reactivity with other heterologous DRV strains in the ELISA. The relative binding to heterologous DRV isolates is expressed as percentage by taking the A405 obtained with DRV S12 in the reaction as 100. Binding was considered as strong if it was more than 50%, significant if it was 25-50%, and negative if it was less than 25%. The results indicated that σB in cell extracts prepared from DEF infected with each heterologous DRV strains was captured by anti-σB antiserum. There are no appreciable differences in the binding of the different MAb's tested. As expected, negative results were obtained using the mock infected DEF (< 25%). Therefore, MAbs 1E5 and 2F7 strongly recognized all tested virus strains, suggesting that epitopes A and B are commonly present among σB of DRV strains and also indicating MAbs 1E5 and 2F7 suitable candidates for the diagnosis of DRV isolates.

## Discussion

The results showed that the antigen preparations containing the expressed His-σB protein of DRV could induce the production of MAbs. After screening and subcloning, the four MAbs directed against His-σB were isolated and characterized. Analysis of immunofluorescence assay indicates that these MAbs bound to the authentic viral protein σB of DRV S12. Thus, the epitopes on the σB recognized by these MAbs were also present on the viral σB of DRV. As for the conformation of His-σB protein in antibody binding, all MAbs bound to the His-σB in its native conformation. When SDS and 2-mercaptoethanol were used to denature the σB-His protein, this binding still remained, indicating that the recognized epitopes were not affected by breaking of disulfide bonds. This led us to suggest that the MAbs binding was conformation independent.

The competitive binding assays were used to determine epitopes of MAbs based on the notion that a MAb binding to a specific site can block the attachment of another MAb to the same site. Two epitopes, A and B, inhibited almost completely the binding of HRP-coupled MAbs recognizing the same epitope, but no competition was obtained among MAbs recognizing epitope A or B. It has not been determined if the epitope A or B involves in any biological functions, but they are highly conserved among DRV strains as the MAbs could recognize both epitopes on all virus strains tested.

DRV σB protein has one basic stretch (KKVSHYR, amino acids 287-293), which is required for dsRNA-binding activity [21]. Thus, to illustrate whether the σB responsible for dsRNA binding or not is currently being investigated by the preparation of deleted mutant proteins of DRV σB along with MAbs described here.

All of these MAbs could successfully detect native-form σB protein in infected cells, as well as in viral particles. Thus, these MAbs may be useful in the development of sensitive methods used for the diagnosis of DRV, such as immunoblot assay, immunofluorescence assay and antigen-capture ELISA. Antigen-capture ELISA using anti-virus antibodies has been an ideal choice for large screening, quantitative analysis of viral antigen or virus titer because of its high sensitivity, reproducibility, and automation.

In this study, we generated four positive clones secreting specific and highly reactive antibodies against DRV σB protein in order to develop diagnostic methods. The results reveal that the MAbs capture ELISA clearly differentiates the samples between the DRV- and mock-infected as demonstrated by absorbance values, suggesting that non-specific reactions could be markedly reduced in the MAb capture ELISA. Both MAbs (1E5 and 2F7) recognize DRV σB at different sites which are highly conserved in all DRV strains tested in the present study. Thus, MAbs (1E5 and 2F7) capture ELISA seems acceptable as a screening method for the detection of DRV in infected birds in future.

## Conclusion

In summary, the results of this experiment provide important information about the monoclonal antibodies against Muscovy duck reovirus σB protein. Especially the monoclonal antibodies could contribute for the development of a MAb capture ELISA for rapid detection of DRVs. In addition, it showed that the σB protein was located in the cytoplasma of infected cells by immunofluorescence assay with MAbs. Although virus isolation and RT-PCR are reliable way for detection of DRV infection, these procedures are laborious, time consuming, and requiring instruments. These obvious diagnosis problems highlight the ongoing demand of rapid, reproducible, and automatic methods for the sensitive detection of DRV.

## Abbreviations

DRV: Muscovy duck reovirus; MAb: monoclonal antibody; ELISA: enzyme-linked immunosorbent assay; RT-PCR: reverse-transcription polymerase chain reaction; dsRNA: double-stranded RNA; DEF: duck embryo fibroblasts.

## Competing interests

The authors declare that they have no competing interests.

## Authors' contributions

YZ and HLC were responsible for the research design and writing of this manuscript. ML, XDC, YW, YFL, and YFW, and NS performed monoclonal antibody preparation and characterization, cloning and sequencing of σB of DRV S12 isolates. All authors read and approved the final manuscript.
